# Relationship between reactive group chemistry and printing properties of heterofunctional reactive dyes via screen printing

**DOI:** 10.1038/s41598-023-33819-3

**Published:** 2023-05-04

**Authors:** Umme Habibah Siddiqua, Shaukat Ali, Aasma Tufail, Mansour K. Gatasheh, Luqman Riaz, Muhammad Wahab Yasir

**Affiliations:** 1 Department of Chemistry, University of Jhang, Jhang, 3500 Pakistan; 2 Department of Chemistry, University of Agriculture, Faisalabad, 38040 Pakistan; 3 Division of Science and Technology, Department of Botany, University of Education, Lahore, Pakistan; 4 Department of Biochemistry, College of Science, King Saud University, P.O.Box 2455, Riyadh, 11451 Saudi Arabia; 5 College of Life Sciences, Henan Normal University, Xinxiang, 453007 Henan China; 6 Department of Environmental and Sustainable Engineering, State University of New York at University at Albany, Washington Ave., Albany, NY 12222 USA

**Keywords:** Chemistry, Materials science

## Abstract

Screen printing of cotton fabric using newly synthesized azo reactive dyes was carried out in the present study. Functional group chemistry and its effect on the printing properties of cotton fabric by varying the nature, number and position of reactive groups of synthesized azo reactive dyes (D1–D6) was studied. Different printing parameters (Temperature, alkali and urea) and their effect was explored on the physicochemical printing properties e.g., fixation, color yield, and penetration of the dyed cotton fabric. Data revealed that dyes with more reactive groups and having linear and planar structures (D-6) showed enhanced printing properties. Spectraflash spectrophotometer was used to evaluate the colorimetric properties of screen-printed cotton fabric and results showed superb color buildup. Printed cotton samples displayed excellent to very good ultraviolet protection factor (UPF). Presence of sulphonate groups and excellent fastness properties may entitle these reactive dyes as commercially viable for urea free printing of cotton fabric.

## Introduction

Printing is the coloration process of creating spectacular motifs and smashing color effects on cellulosic fabric having widespread applications in garment industry, home textile and composite materials^1–3^. Reactive dyes for their wide variety of stunning shades, outstanding fastness properties, simplicity of application methods and forming permanent covalent bonding are more popular for cellulosic fabric printing^4–6^. There is an important role of functional group chemistry and dye structure in the proper dye fiber linkage. The nature, number, and position of the reactive functional groups in the dye molecule have a distinctive impact on printing properties^3,7,8^. Coplanar, linear, sterically less hindered structures having more functional groups are key factors in attaining good quality printing with excellent color yield^9–11^. Fiber reactive dyes comprising of heterofunctional reactive groups have better color yield and greater fixation as compared to reactive dyes containing monofunctional reactive groups^12^ and are more suitable for printing because they exhibit high solubility, high diffusion, low affinity and high printing paste ability^13–15^. Moreover, for practical application of dye on cellulosic fabric there are some technical problems including diffusion and solubility of dyes in the printing paste^7,16^. Meantime, the high substantivity of dyes on the cotton fabric is responsible for patchy printing and the difficult washout process of unfixed dye on printed fabric^17–19^. So, fiber reactive dyes particularly with better and enhanced printing properties are still in demand. Reactive dyes having high solubility in the printing paste with sufficient affinity and substantivity are requisites for the printing of cellulosic fabric at the same time^20–22^. Moreover, dyes should have suitable tinctorial yield, great reactivity, and less dye effluent with the highest degree of fixation. In the current study, six hetero-functional reactive dyes that were designed and synthesized in our previous studies by varying the nature, position and number of functional groups were selected for screen printing of cellulosic fabric to explore the effect of reactive group chemistry on the printing properties. Different process parameters (temperature, urea, alkali) were also optimized to enhance color yield, fastness properties and to maximally reduce the dye discharge in the wastewater, to ensure the sustainable environmental development. Reactive dyes containing both monohlorotriazine (MCT) and sulphatoethylsulphone (SES) reactive moieties with sulphonate groups were used for achieving printed samples with proficient printing and aesthetic properties than dyes containing only one type of reactive group by increasing their solubility in printing paste.

## Experimental

### Materials

A bleached, mercerized 100% cotton fabric (having 124 and 84 ends per inch (epi) and picks per inch (ppi) respectively) with plain weave and areal density of 97.8 g/m^2^ was used in the present research work for the application of reactive dyes through the screen-printing technique. Solid raw materials used for dye synthesis including 2,4,6 trichloro-1,3,5 triazine, 2[(4-Aminophenyl) sulfonyl] ethyl hydrogen sulfate and 1-amino-8-hydroxynaphthalene-3,6-disulphonic acid were taken from Sandal Dyes and Chemical, (Pvt.) Ltd. Faisalabad, Pakistan. Analytical grade printing auxiliaries including urea, sodium alginate, NaHCO_3_, perlavin PAM detergent were obtained from BASF Germany.

### Synthesis of heterofunctional reactive dyes

**Scheme 1.**
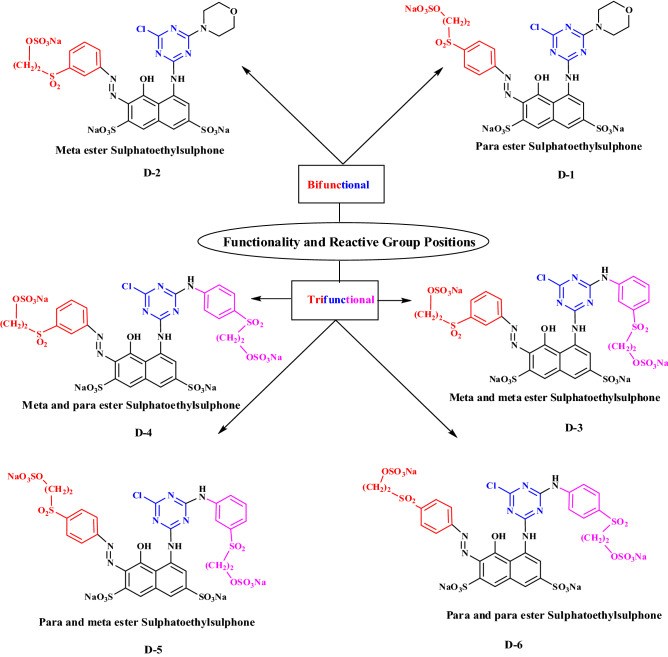
Chemical structures of Heterofunctional azo reactive dyes.

Condensation, diazotization of sulphatoethylsulphone and H-acid coupling reactions were carried out for the synthesis of heterofunctional reactive dyes (D-1 to D-6) as described in the previous study^3^. The structures of the synthesized dyes are given in Scheme 1. New dye structures were characterized and confirmed through ultraviolet–visible, Fourier transform infrared and electrospray ionized mass spectrometry analytical techniques as discussed in previous study^13^. Characterization results are also given in supplementary data.

#### D-1

Physical state: powder, Colour: Red, Yield: 85%, Molecular formula: C_25_H_17_N_7_O_14_S_4_ClNa_3_, UV/Vis spectra in water: 459 nm, IR (KBr cm^−1^): 1543.57 (N=N Stretching), 1049.17–1201.76 (–S=O Stretching), 3417.87 (–NH Stretching), 1489.21 (–C–N Stretching), 740–799 (–C–Cl Stretching), ESI–MS: *m/z* = 413.42 [M-2Na] were detected.

#### D-2

Physical state: Powder, Color: Red, Yield: 82%, Molecular formula: C_25_H_17_N_7_O_14_S_4_ClNa_3_, UV/Vis spectra in water: 462 nm, IR (KBr cm^−1^): 1573.91 (N=N Stretching), 1193–1050 (–S=O Stretching), 3395.83 (–NH-Stretching), 1379.83 (–C–N Stretching), 740–799 (–C–Cl Stretching), ESI–MS: *m/z* at 268.75 [M-3Na] were detected.

#### D-3

Physical state: Powder, Color: Red, Yield: 78%, Molecular formula: C_29_H_22_ClN_7_Na_4_O_19_S_6_, UV/Vis spectra in water: 477 nm, IR (KBr cm^−1^): 1573.92 (N=N Stretching), 1050.51 (–S=O Stretching), 1195 (–SO_3_ Stretching), 1409.15 (–C–N Stretching), 742–801 (–C–Cl Stretching), ESI–MS: *m/z* = 522.50 [M-2Na] were detected.

#### D-4

Physical state: powder, Color: Red, Yield: 79%, Molecular formula: C_29_H_22_ClN_7_Na_4_O_19_S_6_, UV/Vis spectra in water: 475 nm, IR (KBr cm^−1^): 1574.19 (N=N Stretching), 1049.59 (–S=O Stretching), 1201–1135 (–SO_3_ Stretching), 1410.19 (–C–N Stretching), 738–801 (–C–Cl Stretching), ESI–MS: *m/z* = 522.50 [M-2Na] were detected.

#### D-5

Physical state: Powder, Color: Red, Yield: 75%, Molecular formula: C_29_H_22_ClN_7_Na_4_O_19_S_6_, UV/Vis spectra in water: 475 nm, IR (KBr cm^−1^): 1573.80 (N=N Stretching), 1050.21 (–S=O Stretching), 1201–1134 (–SO_3_ Stretching), 2956.34 (–C–H Stretching), 737.50–995.58 (–C–Cl Stretching), ESI–MS: *m/z* = 249.83 [M-4Na] were detected.

#### D-6

Physical state: Powder, Color: Red, Yield: 80%, Molecular formula: C_29_H_22_ClN_7_Na_4_O_19_S_6_, UV/Vis spectra in water: 480 nm, IR (KBr cm^−1^): 1573.92 (N=N Stretching), 1051.34 (–S=O Stretching), 1139.08 (–SO_3_ Stretching), 1433.07 (C=C Stretching), 996.17 (–C–Cl Stretching), ESI–MS: *m/z* = 249.83 [M-4Na] were detected.

### Screen printing

#### Printing paste preparation

Printing paste was prepared using auxiliaries including 8% urea and 6% NaHCO_3_ (alkali) and 4% sodium alginate as thickening agent for the application of each dye on the cotton fabric. Each dye Stock solution (3% w/w) was employed in the printing paste preparations^23^.

#### Printing technique

Screen printing technique using Flat Bed Screen Printing Machine Tsujii Printing Machine Mfg. Co. Ltd. Osaka/Japan (SP-300AR) was carried out for the application of printing paste on cotton fabric at the viscosity of 2300 cps using a laboratory-scale printing machine. Steaming of printed samples using Mathis laboratory steamer CH-8155 (Wenner Mathis Co., Switzerland) was carried out for 15 min at 105 °C. After printing unfixed dye and thickener were detached from the printed cotton sample by rinsing through cold then hot water until the bleeding stopped; perlavin PAM (2 g/L) was used 15 min for soaping at the boil and finally dry in the open air. Fastness properties were determined employing different fastness testing and spectraflash spectrophotometer Color Eye, Gretagmacbeth (7000 A) was used to evaluate the colorimetric data of the printed fabric^24^.

### Evaluation of fixation, penetration and colorimetric data of printed samples

K/S values of the printed cotton samples were determined before and after washing treatments which reflects the fixation percentage of dye covalently attached to the fabric. Spectraflash spectrometer was used for the determination of the color strength of printed samples at their particular λ_max_. Calculations of fixation ratio were carried out using Eq. (1) ^25^.

1 $$\mathrm{\%F}=\frac{{(K/S)}_{2}}{{(K/S)}_{1}} \times 100,$$

where (K/S)_1_ and (K/S)_2_ denotes the color strengths of the printed cotton samples before and after washing, respectively.

The penetration percent (P %) was calculated according to Eq. (2).

2 $$\mathrm{\%P}=\frac{({K/S)}_{b}}{{(K/S)}_{f}} \times 100,$$

where (K/S)_b_ and (K/S)_f_ represent the non-printed back face and printed front face values of K/S of fabric, respectively. If penetration percentage higher than it shows the greater penetration of the dye into the fabric^26^.

Printed samples were subjected to LCH and CIELAB system for the evaluation of colorimetric data L*, a*, b*, C* and h* representing lightness/darkness, redder/greener, redder/greener, chroma, and hue, respectively for one color determination^27^. Spectraflash spectrophotometer 7000 A (Color Eye, Gretagmacbeth) was used for the determination of color parameters in National Textile University Faisalabad. The colorimetric data of the printed fabric is given in Table 1.

**Table 1:** Colorimetric values of the printed samples.

Dye code	L*	a*	b*	C*	hº
D-1	50.03	45.88	− 4.37	46.09	354.56
D-2	51.25	43.01	− 3.98	39.06	0.6
D-3	43.53	52.19	0.78	52.19	3.95
D-4	41.25	53.00	2.45	52.56	2.68
D-5	41.17	52.31	2.42	52.36	2.65
D-6	39.38	53.07	5.22	53.32	5.60

### UPF and fastness properties of the printed fabric

Ultraviolet protection factor (UPF) and fastness properties of the printed samples were evaluated using AATCC TM 183 and different ISO test methods respectively. ISO-105 CO3 test method was used for assessing the washing fastness, ISO 105-X12 test method for Crock fastness, ISO 105-B02 for light fastness, ISO-E04 for perspiration fastness, and ISO 105-E03 test method for evaluating the fastness to chlorinated water of printed samples.

## Results and discussion

### Effect of temperature on printing properties of reactive dyes

**Figure 1:**
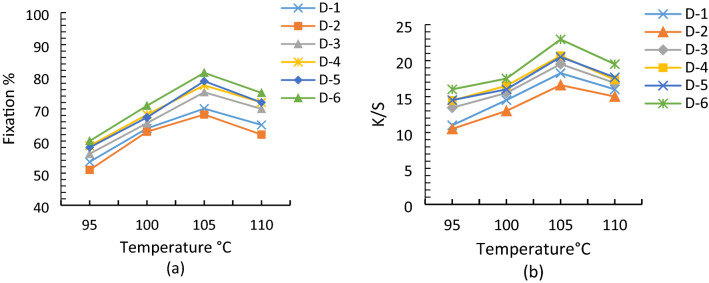
Effect of temperature on the (**a**) fixation % and (**b**) color yield of reactive dyes.

Reactive dyes first adsorbed on the fabric surface through an ionic bond. Once assembled, the ionic bond was then converted to a covalent bond at required optimum temperatures^28,29^. The optimum temperature for steaming of printed cotton samples was 105 °C at which maximum penetration, fixation, and color yield was obtained^30^ as shown in Fig. 1. Rate of dye fixation decreased at high temperature because activation energy for dye-fiber interaction is 9.2–15.8 kcal while for dye-water interaction is 16.4–26.2 kcal^28^. So, at higher temperature than optimum the dye-water reaction rate increased which is responsible for dye hydrolysis and in turn poor washing fastness. Dye hydrolysis also increases the loss of dye in the effluent which caused potential pollution problems. At low temperature dye molecules mobility could be low which required more printing time. Klančnik^32^ also studied the effect of temperature on the dyeing behaviour of the reactive dyes^31,32^.

### Urea influence on the fixation and color yield of reactive dyes

**Figure 2:**
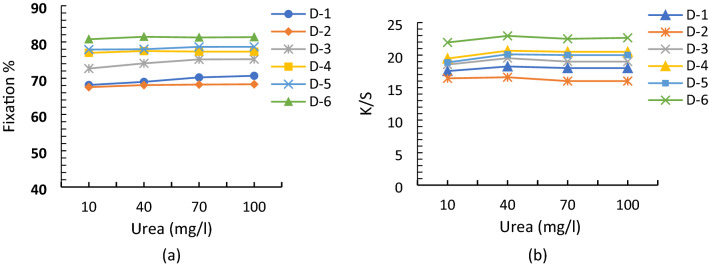
Effect of urea on the (**a**) fixation % and (**b**) color yield of reactive dyes.

The key function of the urea in the printing paste is to facilitate the dye-fiber interaction by reducing the dye aggregation and increasing its solubility in the reaction medium^32^. It also retards the evaporation of water during drying and swelling of cellulosic fabric in the printing process^33,34^. All heterofunctional reactive dyes used during the printing technique were water-soluble, vinylsulphone based, and have sulfonate groups which increased their solubility in printing paste^29,35^. So, color yield and fixation % of the printing samples were not affected greatly by the urea concentration. The effect of urea on the K/S and fixation values of dyes (D-1 to D-6) is shown in Fig. 2. These results demonstrated that urea free dyeing is possible for vinylsulphone based dyes having sulfonate groups in their structures^26,36,37^. Urea poses many environmental and ecological issues due to high nitrogen contents in the printing effluent.

### Alkali influence on the fixation and color yield of reactive dyes

**Figure 3:**
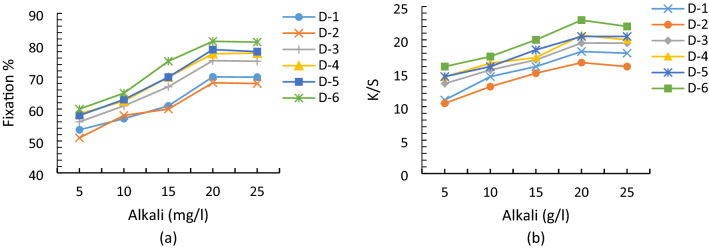
Effect of alkali on the (**a**) % fixation and (**b**) color yield of reactive dyes.

Alkali was very important for screen printing of dyes because sulphatoethylsulphone reactive groups were converted into their active vinylsulphone form through addition reaction which was possible in the presence of alkali and at appropriate pH. Alkali was also necessary for the activation of the cotton material so that fabric pores are in exact orientation in the printing medium for covalent bonding with the dye molecules^38,39^. The effect of alkali on the color yield and % fixation of the printed cotton samples is shown in Fig. 3. It was clear from data that printing properties (K/S and fixation, penetration) were increased with the alkali concentration up to a specific limit. Beyond this limit, printing properties started decreasing because the rate of dye hydrolysis might be increased in the printing medium^40,41^.

### Colorimetric data

**Figure 4:**
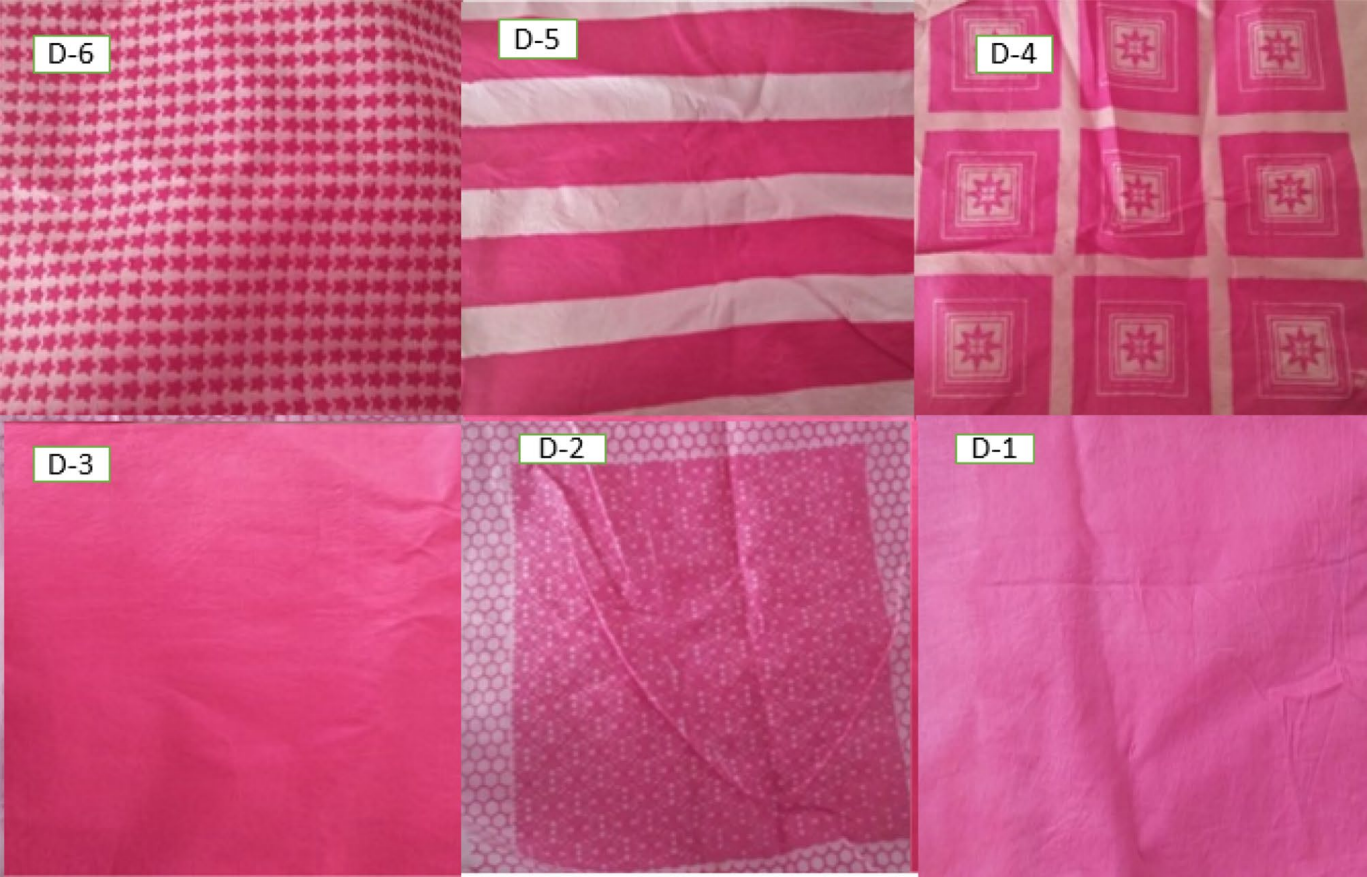
Patterns of screen-printed cotton fabric by heterofunctional azo reactive dyes.

Colorimetric properties of the printed cotton fabric with heterofunctional reactive dyes were obtained through spectraflash spectrophotometer and results are presented in Table 1. Different patterns of screen printing were employed on cotton fabric by six heterofunctional azo reactive dyes which are represented by Fig. 4. The h° (hue angle) values from the colorimetric data illustrated purple-red shades for all dyes and K/S values suggested high color strength on printed cotton fabric. All printed samples have brighter (higher C* values) and darker shades (low L* values) demonstrating that the printing technique was suitable for the synthesized heterofunctional reactive dyes. Trifunctional reactive dyes (D-3 to D-6) have low L* values and higher a* values representing darker shades and redder tones on printed fabric. Conversely, bifunctional reactive dyes (D-1 and D-2) have lighter (high L* value) shades and gave a bluer tone on the cotton fabric (higher − b* values). Colorimetric values from a* and b* represented the much redder and less yellowish tones of the printed samples. Data from K/S values revealed that the color yield of trifunctional reactive dyes was higher as compared to bifunctional reactive dyes.

### UPF and color fastness of the printed cotton samples

UPF factor of the control (non-printed) fabric was 3.5. Results from Table 2 showed that the UPF factor of the printed fabrics was excellent to very good at 3% dye shade. UPF value increased as dye fixation and penetration increased on the cotton fabric and results showed that D-6 has highest UPF value having highest color yield^35,42^. This is related to its more planar and sterically less hindered trifunctional structure responsible for highest penetration and fixation of dye on the cotton fabric as reactive groups are present at the para positions in D-6. D-2 has lowest UPF value which may be attributed to its bifunctionality and meta position of the reactive groups.

**Table 2:** UPF and Color Fastness of the printed cotton fabric.

Dye Code	UPF mean	Washing fastness		Rubbing fastness		Light fastness	Perspiration fastness	Chlorinated fastness
		Shade change	Staining	Shade change	Staining			
D-1	31	4	4	4	3–4	4–5	3–4	3
D-2	28	3–4	4	4	3–4	4–5	3–4	3
D-3	45	4	4	4	3–4	5	4	3–4
D-4	47	4	4	4	4	5	4	3–4
D-5	47	4	4	4	4	5	4–5	3–4
D-6	50	4	4	4	4	5	4–5	3–4

Data outcomes from Table 2 showed good (4) washing fastness of printed cotton samples which may be credited to dye molecules fixation through covalent bond formation with the cotton samples^43^. Crock fastness was also good of the printed cotton samples. Bifunctional reactive dyes have moderate rubbing as compared to trifunctional reactive dyes. Hydrolyzed dye on the fiber surface was responsible for staining during rubbing fastness and results from Table 2 showed that contents of hydrolyzed dye was minimum because maximum dye molecules fixed on printed samples dye to strong covalent bonding. Good to excellent (4–5) light fastness was observed from results which may be attributed to the fact that H-acid azo dyes chromophores can undergo to azo-hydrazone tautomerism making H-acid azo dyes stable to photoreduction^44^. Presence of heterofunctional reactive groups (triazine and vinylsulphone) in newly synthesized azo dyes makes them stable to both alkaline and acidic conditions showing good perspiration fastness^36^. Fastness to chlorinated water showed moderate (3) results for bifunctional dyes while moderate to good (3–4) results were obtained for the trifunctional reactive dyes.

### Reactive group chemistry and printing properties

There is an important role of functional group chemistry and dye structure in the proper dye fiber linkage. The heterofunctional reactive groups are responsible for greater affinity of dye molecule towards cellulosic fiber. Coplanar, linear, sterically less hindered structures having more functional groups are key factors in attaining good quality printing with excellent color yield. In this context, in present study printing properties were investigated by changing the number and positions of reactive groups during screen printing of cellulosic fabric^45^. Reactive dyes attached and fixed on the cotton fabric by forming a permanent covalent bond between the fiber hydroxyl and dye reactive groups^46–48^. As the reactive group's number increased, the extent of bonding increased which ultimately increased fixation and color yield. The increasing order of printing properties D6 ˃ D-5 ˃ D-4 ˃ D-3 ˃ D-2 ˃ D-1 showed that trifunctional reactive dyes revealed enhanced printing properties (penetration, fixation, UPF value and color yield) as compared to bifunctional reactive dyes (Table 3). Dye structures should be more planar and sterically less hindered so that effective dye fiber interaction takes place, which consequently increased the dye penetration and fixation in the cellulosic fabric pores^3,46,49^. As penetration and fixation increased, the color yield of printed fabric automatically increased^50,51^. D-6 having more planar and sterically less hindered structure (reactive groups present at para positions) had increased color yield, higher fixation, penetration and UPF value as compared to D-1, D-2, D-3, D-4, and D-5. The penetration and fixation of dyes increased into the cotton fabric by increasing the number of reactive groups because more reactive sites were available to fiber substrate for binding which leads to high color yield^52–54^. Therefore, trifunctional reactive dyes have high UPF value than bifunctional reactive dyes as UPF value depends on color strength^27,43^. Synthesized dyes were vinylsulphone based having sulfonate groups in their structures so they have a higher solubility in water and consequently in the printing paste which makes them more suitable for urea free printing^26^. Different patterns of screen-printed cotton fabrics by heterofunctional reactive dyes are shown in Fig. 4.

**Table 3:** Printing properties of heterofunctional reactive dyes on cotton fabric.

Dye code	λ_max_ (nm)	K/S	F (%)	P (%)
D-1	459	18.25	70.10	6.43
D-2	462	16.59	68.25	5.25
D-3	477	19.52	75.19	9.33
D-4	475	20.50	77.35	10.22
D-5	475	20.67	78.65	10.45
D-6	480	22.95	81.24	12.25

## Conclusion

The current study showed that reactive group chemistry significantly influences the printing properties of cotton fabric. The optimum temperature for steaming of printed cotton samples was 105 °C which prominently affects the printing properties of reactive dyes. Six heterofunctional reactive dyes were sensitive to alkali but urea concentration had no noticeable effect on the fixation and K/S values. All dyes showed excellent to good fastness properties onto the printed cellulosic fabric which may be attributed to the permanent covalent bonding of reactive dyes. Trifunctional reactive dyes (D-3 to D-6) have enhanced printing properties as compared to bifunctional reactive dyes (D-1 and D-2). The penetration and fixation of dyes increased into the cotton fabric as the number of reactive groups and planarity increased and steric hindrance decreased in the dye structures which consequently increased the K/S value. UPF of the printed samples was excellent to very good. Vinylsulphone reactive dyes having sulfonate groups in their structure have high solubility in printing paste. Therefore, it is suggested that these six reactive dyes could be commercially practicable for urea free printing of cellulosic fabric.

## Supplementary Information

Supplementary Information.

## Data Availability

The authors confirm that the data supporting the findings of this study are available within the article including supplementary data.
