# Supplementary material for: Relationship between reactive group chemistry and printing properties of heterofunctional reactive dyes via screen printing
Source: Sci Rep. 2023 May 4;13:7259. doi: 10.1038/s41598-023-33819-3 (PMC10160041; doi:10.1038/s41598-023-33819-3)
Supplement: Supplementary file 1 — Supplementary Information. [file 41598_2023_33819_MOESM1_ESM.docx]

**Supplementary Data**

**UV/Vis spectra of heterofunctional reactive dye**


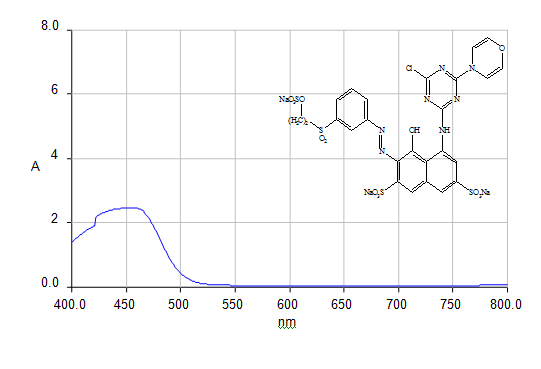


**Figure S1.** UV/Vis spectra of reactive azo dye D-1


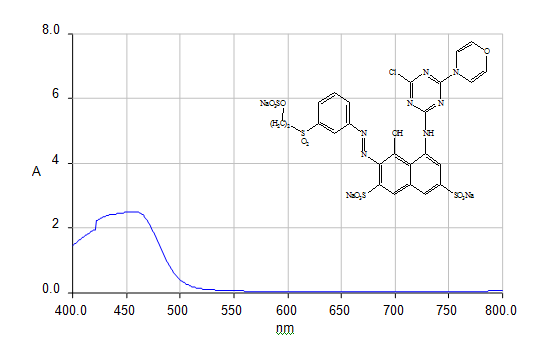


**Figure S2.** UV/Vis spectra of reactive azo dye D-2


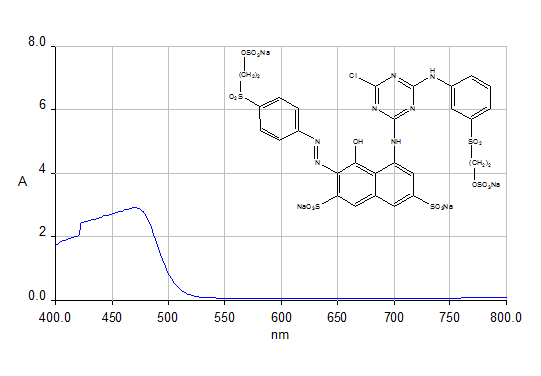


**Figure S3.** UV/Vis spectra of reactive azo dye D-3


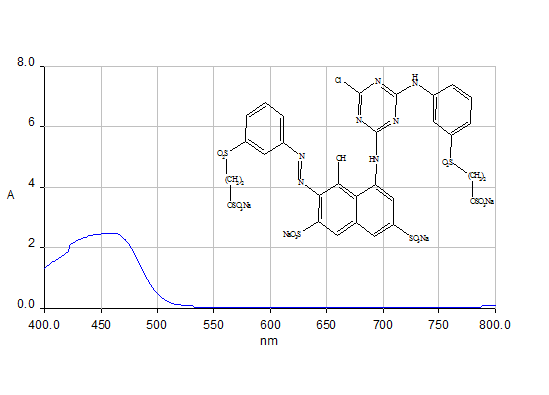


**Figure S4.** UV/Vis spectra of reactive azo dye D-4


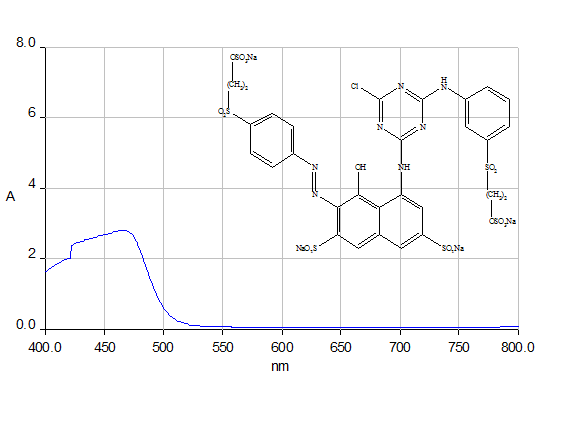


**Figure S5.** UV/Vis spectra of reactive azo dye D-5


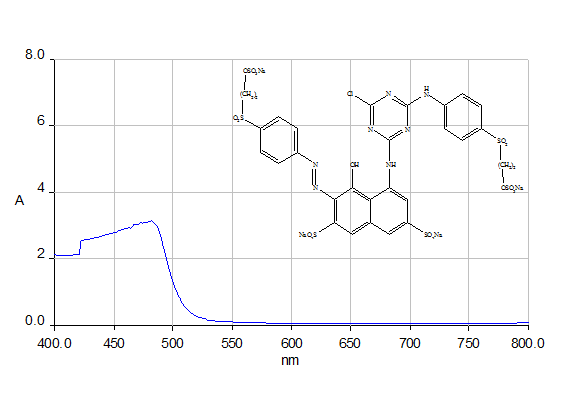


**Figure S6.** UV/Vis spectra of reactive azo dye D-6

FT-IR spectrum of heterofunctional reactive dye
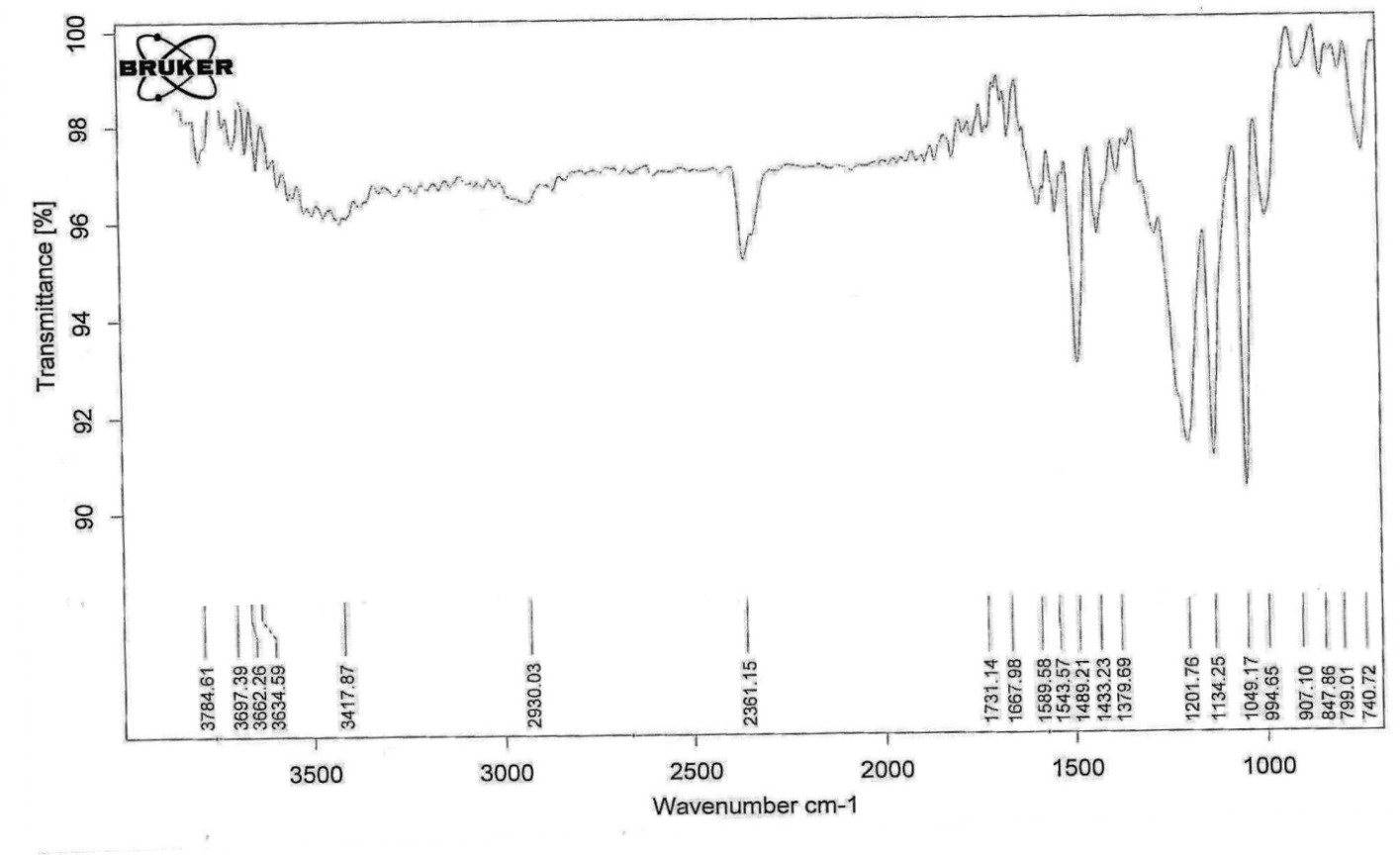
 **Figure S7.** FT-IR spectrum of heterofunctional reactive dye D-1

**Table S1.** Stretching and bending frequencies of characteristics functional groups present in dye D-1

| **Characteristic Peak (cm^-1^)** | **Assignment** | **Functional group** |
| --- | --- | --- |
| 3417.87 | N-H Stretching | -NH- |
| 2930.03 | C-H Stretching | -CH= |
| 1543.57 | N=N Stretching | -N=N- |
| 1489.21 | -C-N Stretching | Triazine ring |
| 1049.17- 1201.76 | -S=O Stretching | -SO_2_ |
| 740-799 | -C-Cl Stretching | Substituted benzene rings |


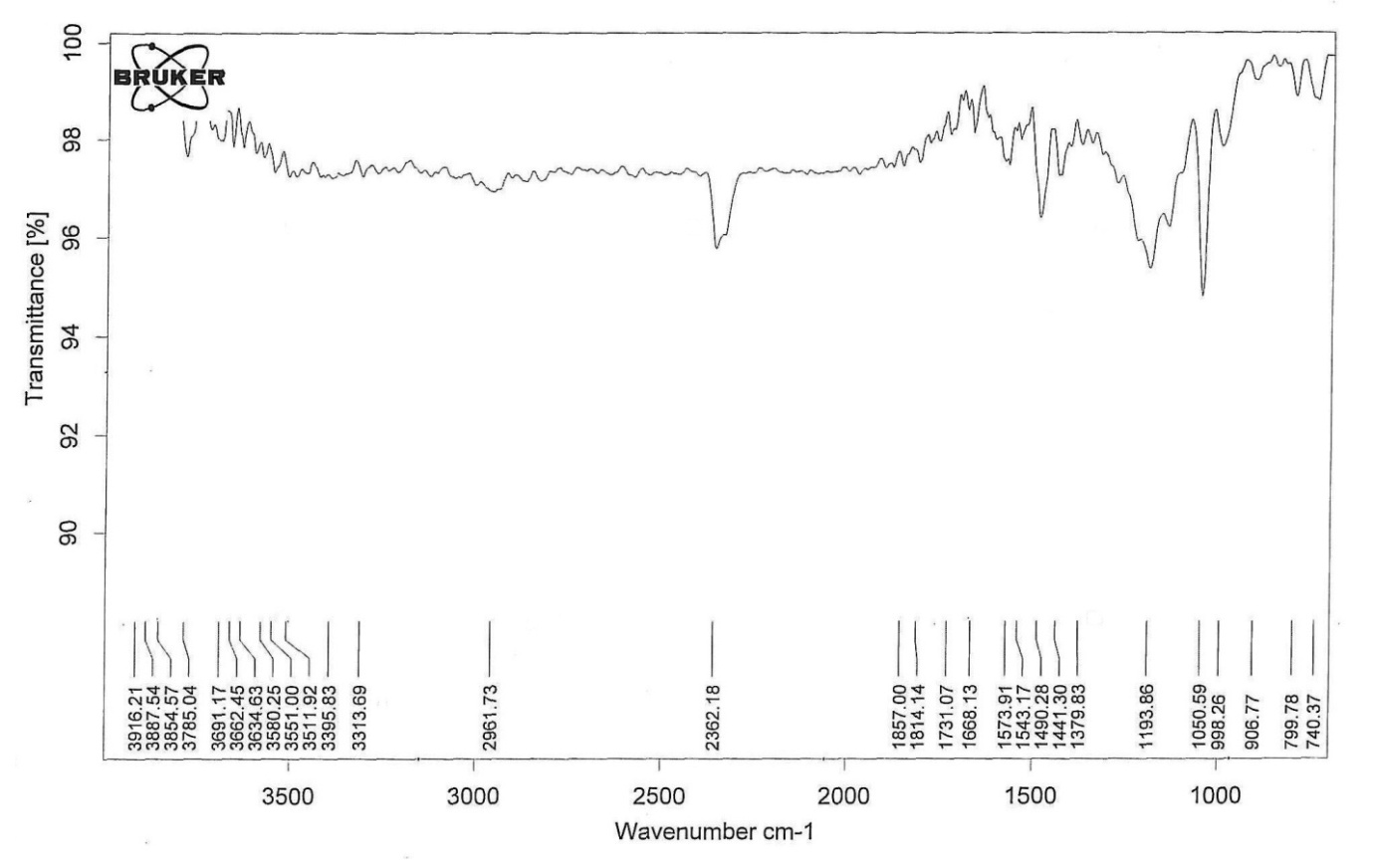


**Figure S8.** FT-IR spectrum of heterofunctional reactive dye D-2

**Table S2.** Stretching and bending frequencies of characteristics functional groups present in dye D-2

| **Characteristic Peak (cm^-1^)** | **Assignment** | **Functional group** |
| --- | --- | --- |
| 3395.83 | N-H Stretching | -NH- |
| 2961.73 | C-H Stretching | -CH= |
| 1573.91 | N=N Stretching | -N=N- |
| 1379.83 | -C-N Stretching | Triazine ring |
| 1193-1050 | -S=O Stretching | -SO_2_ |
| 740-799 | -C-Cl Stretching | Substituted benzene rings |


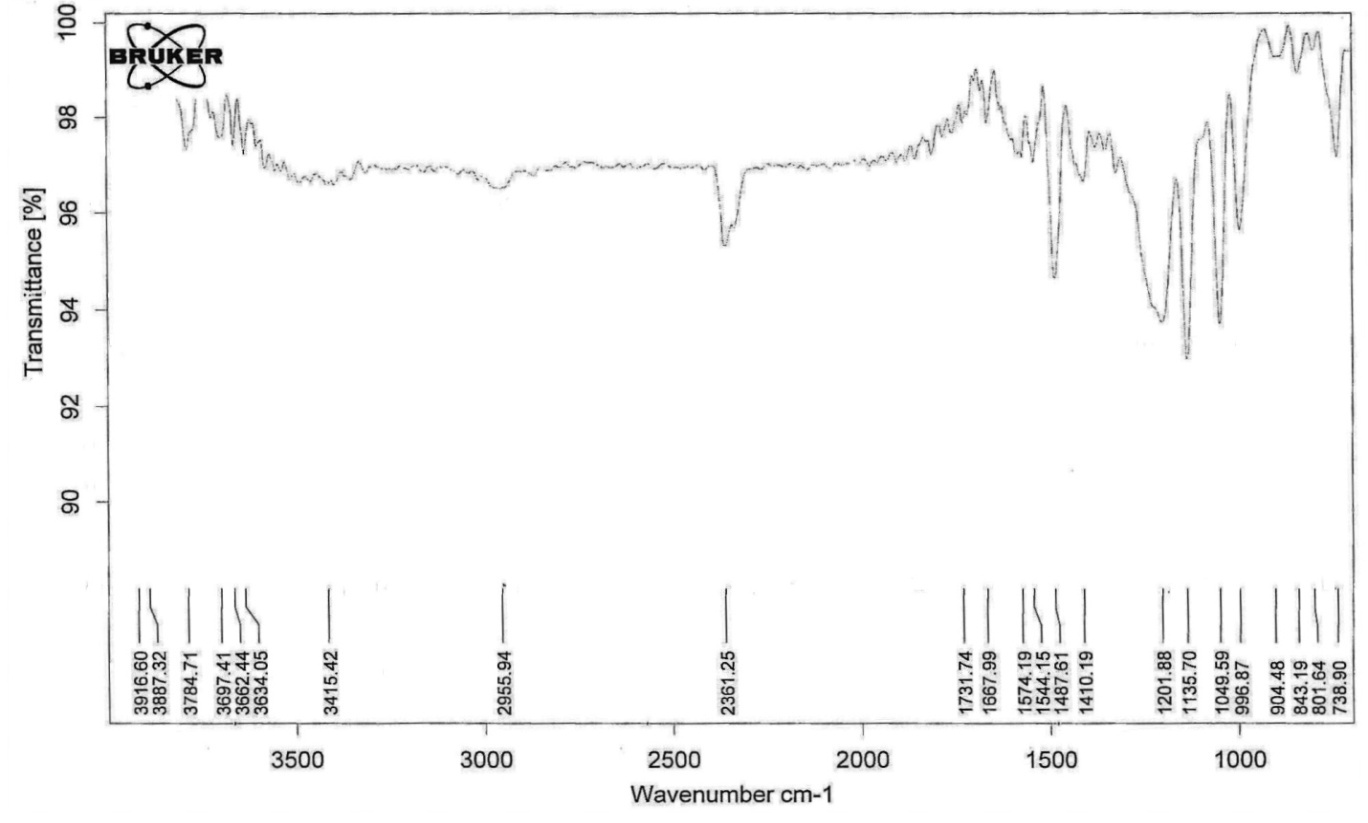


**Figure S9.** FT-IR spectrum of heterofunctional reactive dye D-3

**Table S3.** Stretching and bending frequencies of characteristics functional groups present in dye D-3

| **Characteristic Peak (cm^-1^)** | **Assignment** | **Functional group** |
| --- | --- | --- |
| 3415.42 | N-H Stretching | -NH- |
| 2955.94 | C-H Stretching | -CH= |
| 1574.19 | N=N Stretching | -N=N- |
| 1410.19 | -C-N Stretching | Triazine ring |
| 1201-1135 | -SO_3_ Stretching | -SO_3_H |
| 1049.59 | -S=O Stretching | -SO_2_ |
| 738-801 | -C-Cl Stretching | Substituted benzene rings |


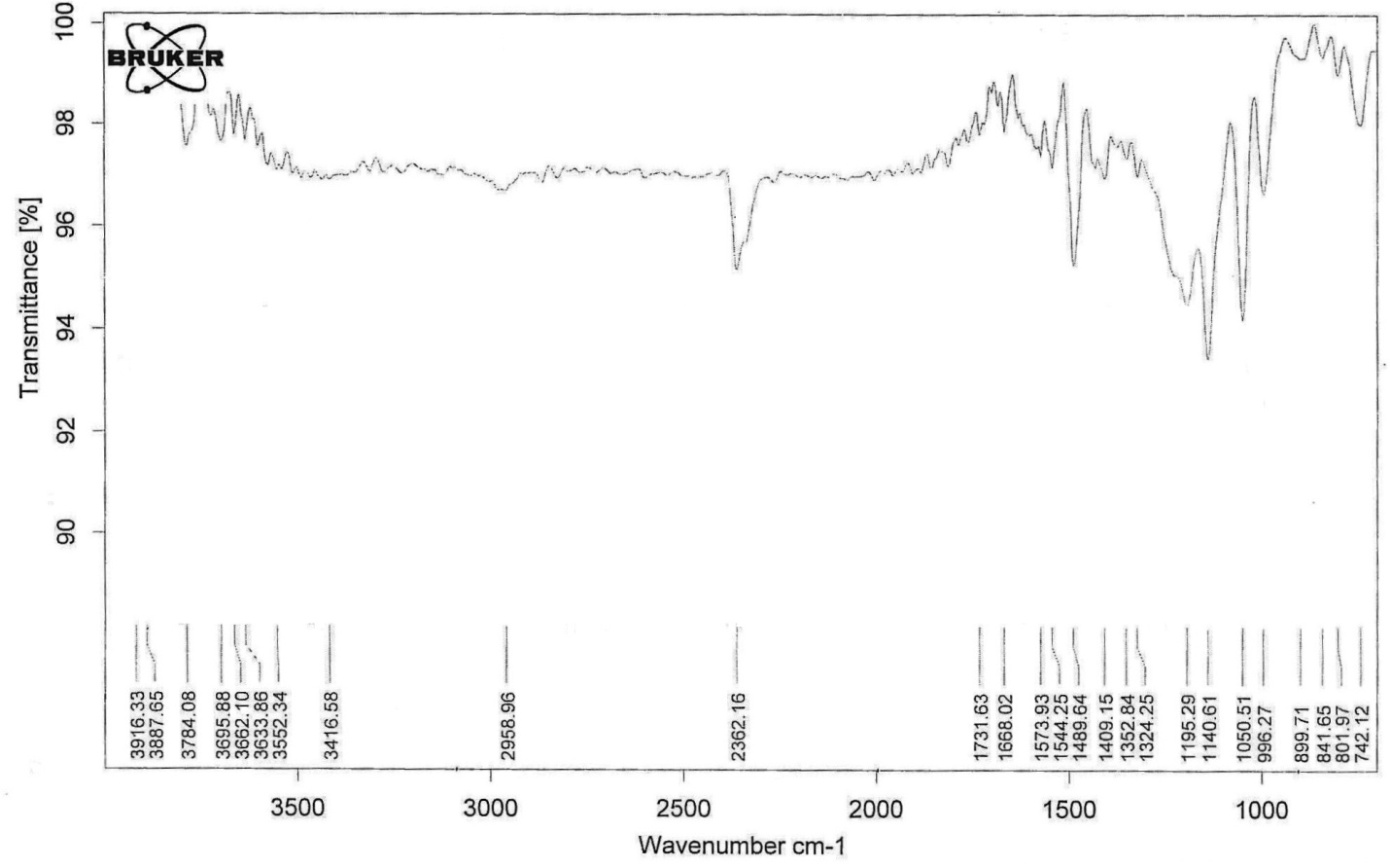


**Figure S10.** FT-IR spectrum of heterofunctional reactive dye D-4

**Table S4.** Stretching and bending frequencies of characteristics functional groups present in dye D-4

| **Characteristic Peak (cm^-1^)** | **Assignment** | **Functional group** |
| --- | --- | --- |
| 3416.58 | N-H Stretching | -NH- |
| 2958.96 | C-H Stretching | -CH= |
| 1573.93 | N=N Stretching | -N=N- |
| 1409.15 | -C-N Stretching | Triazine ring |
| 1195-1140 | -SO_3_ Stretching | -SO_3_H |
| 1050.51 | -S=O Stretching | -SO_2_ |
| 742-801 | -C-Cl Stretching | Substituted benzene rings |


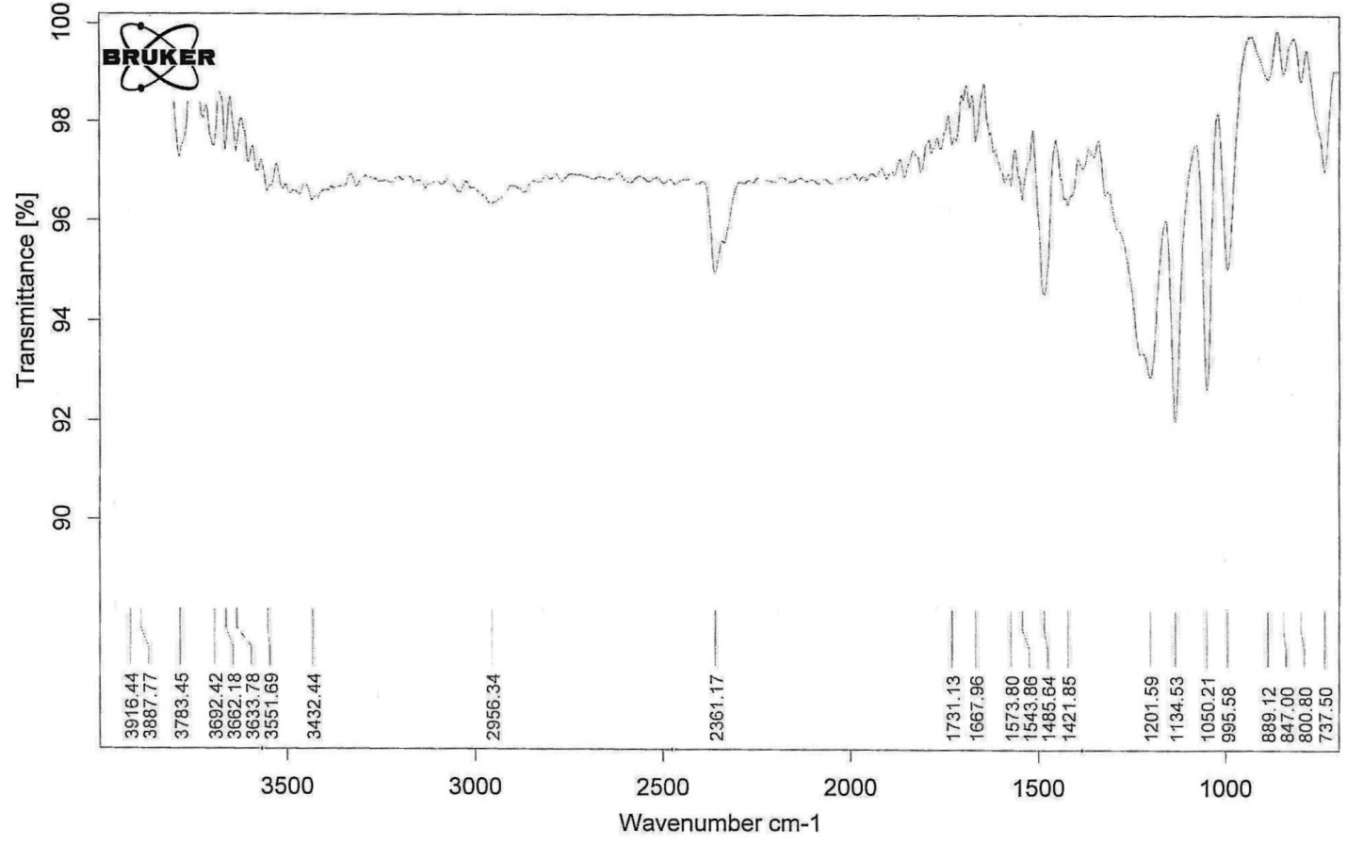


**Figure S11.** FT-IR spectrum of heterofunctional reactive dye D-5

**Table S5.** Stretching and bending frequencies of characteristics functional groups present in dye D-5

| **Characteristic Peak (cm^-1^)** | **Assignment** | **Functional group** |
| --- | --- | --- |
| 3432.44 | N-H Stretching | -NH- |
| 2956.34 | C-H Stretching | -CH= |
| 1573.80 | N=N Stretching | -N=N- |
| 1421.85 | C=C Stretching | Aromatic ring |
| 1201-1134 | -SO_3_ Stretching | -SO_3_H |
| 1050.21 | -S=O Stretching | -SO_2_ |
| 737.50,995.58 | -C-Cl Stretching | Substituted benzene rings |


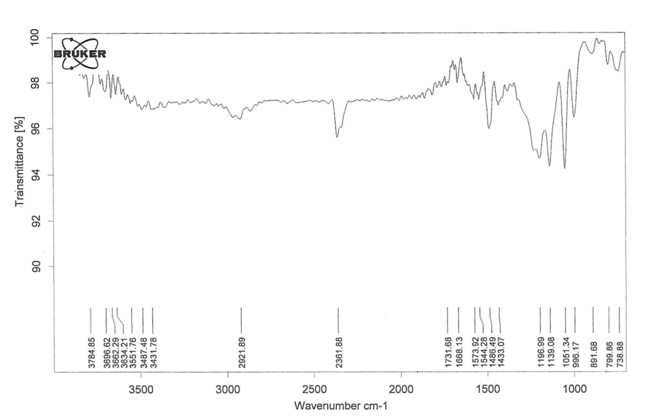


**Figure S12.** FT-IR spectrum of heterofunctional reactive dye D-6

**Table S6.** Stretching and bending frequencies of characteristics functional groups present in dye D-6

| **Characteristic Peak (cm^-1^)** | **Assignment** | **Functional group** |
| --- | --- | --- |
| 3487.48 | N-H Stretching | -NH- |
| 2921.98 | C-H Stretching | -CH= |
| 1573.92 | N=N Stretching | -N=N- |
| 1433.07 | C=C Stretching | Aromatic ring |
| 1139.08 | -SO_3_ Stretching | -SO_3_H |
| 1051.34 | -S=O Stretching | -SO_2_ |
| 738.88, 996.17 | -C-Cl Stretching | Substituted benzene rings |

ESI-MS spectrum of the heterofunctional reactive dyes Dye

**Figure S13.** ESI-MS spectrum of the Dye D-1

**Figure S14.** ESI-MS spectrum of the Dye D-2

**Figure S15.** ESI-MS spectrum of the Dye D-3

****  **Figure S16.** ESI-MS spectrum of the Dye D-4

**Figure S17.** ESI-MS spectrum of the Dye D-5

**Figure S18.** ESI-MS spectrum of the Dye D-6
